# Bis{2-[bis­(2-hy­droxy­eth­yl)amino]­acetato-κ^3^ *O*,*N*,*O*′}zinc(II)

**DOI:** 10.1107/S1600536810043217

**Published:** 2010-10-30

**Authors:** Kong Mun Lo, Seik Weng Ng

**Affiliations:** a Department of Chemistry, University of Malaya, 50603 Kuala Lumpur, Malaysia

## Abstract

In the crystal structure of the zinc(II) complex of bicine, [Zn(C_6_H_12_NO_4_)_2_], the deprotonated amino acid *O*,*N*,*O*′-chelates to the metal atom through a carboxyl­ate O atom, a hy­droxy O atom and the N atom, the three atoms occupying *fac* positions of the distorted octa­hedron surrounding the metal atom. The metal atom lies on a center of inversion. The uncoordinated carboxyl­ate O atom is hydrogen bonded to the hy­droxy groups of adjacent mol­ecules, these two hydrogen bonds leading to the formation of a three-dimensional network.

## Related literature

For the isostructural cobalt(II) analog, see: Zhao & Liu (2010 ▶).
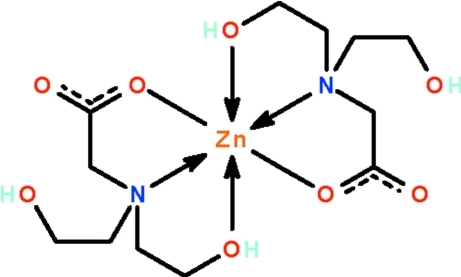


## Experimental

### 

#### Crystal data

[Zn(C_6_H_12_NO_4_)_2_]
*M* *_r_* = 389.70
Monoclinic, 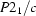

*a* = 9.7863 (7) Å
*b* = 11.3715 (8) Å
*c* = 7.3462 (5) Å
β = 109.1495 (8)°
*V* = 772.28 (9) Å^3^
*Z* = 2
Mo *K*α radiation
μ = 1.64 mm^−1^
*T* = 100 K
0.25 × 0.20 × 0.15 mm

#### Data collection

Bruker SMART APEX diffractometer
Absorption correction: multi-scan (*SADABS*; Sheldrick, 1996 ▶) *T*_min_ = 0.685, *T*_max_ = 0.792
7174 measured reflections
1773 independent reflections
1634 reflections with *I* > 2σ(*I*)
*R*_int_ = 0.026

#### Refinement

*R*[*F* ^2^ > 2σ(*F* ^2^)] = 0.021
*wR*(*F* ^2^) = 0.062
*S* = 1.12
1773 reflections
114 parameters
2 restraints
H atoms treated by a mixture of independent and constrained refinement
Δρ_max_ = 0.43 e Å^−3^
Δρ_min_ = −0.35 e Å^−3^

### 

Data collection: *APEX2* (Bruker, 2009 ▶); cell refinement: *SAINT* (Bruker, 2009 ▶); data reduction: *SAINT*; program(s) used to solve structure: *SHELXS97* (Sheldrick, 2008 ▶); program(s) used to refine structure: *SHELXL97* (Sheldrick, 2008 ▶); molecular graphics: *X-SEED* (Barbour, 2001 ▶); software used to prepare material for publication: *publCIF* (Westrip, 2010 ▶).

## Supplementary Material

Crystal structure: contains datablocks global, I. DOI: 10.1107/S1600536810043217/xu5062sup1.cif

Structure factors: contains datablocks I. DOI: 10.1107/S1600536810043217/xu5062Isup2.hkl

Additional supplementary materials:  crystallographic information; 3D view; checkCIF report

## Figures and Tables

**Table 1:** Hydrogen-bond geometry (Å, °)

*D*—H⋯*A*	*D*—H	H⋯*A*	*D*⋯*A*	*D*—H⋯*A*
O3—H3⋯O2^i^	0.84 (1)	1.85 (1)	2.651 (2)	161 (2)
O4—H4⋯O2^ii^	0.83 (1)	1.89 (1)	2.715 (2)	178 (3)

Symmetry codes: (i) 
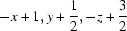
; (ii) 
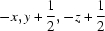
.

**Fig. 1.**
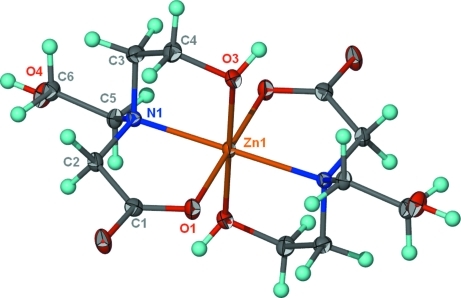
Thermal ellipsoid plot (Barbour, 2001) of Zn(C6H12NO4)2 at the 70% probability level; hydrogen atoms are drawn as spheres of arbitrary radius.

## supplementary crystallographic information

### Comment

In the crystal structure of the cobalt(II) derivative of bicine, the deprotonated aminoacid *N*,*O*,*O*'-chelates to the metal atom through the nitrogen, carboxyl oxygen and hydroxyl oxygen atoms, the three atoms occupying *fac* positions of the octahedron (Zhao & Liu, 2010). The present zinc analog (Scheme I, Fig. 1) is isostructural. The double-bond carboxyl oxygen atom is hydrogen-bond acceptor to the coordinated as well as the free hydroxyl unit of adjacent molecules, these two hydrogen bonds leading to the formation of a three-dimensional network (Table 1).

### Experimental

N,N-Bis(2-hydroxyethyl)glycine (0.94 g, 5.7 mmol) and zinc carbonate (0.36 g, 2.8 mmol) were heated in a 1:1 water:DMSO mixture (100 ml) for 1 h. The solution was filtered; colorless crystals were obtained upon slow evaporation of the filtrate.

### Refinement

Carbon-bound H-atoms were placed in calculated positions (C—H 0.99 Å) and were included in the refinement in the riding model approximation, with *U*(H) set to 1.2*U*(C).

The hydroxy H-atoms were located in a difference Fourier map, and were refined with the O–H distance restrained to 0.84±0.01 Å; its temperature factor was refined.

### Crystal data

**Table:**

[Zn(C_6_H_12_NO_4_)_2_]	*F*(000) = 408
*M**_r_* = 389.70	*D*_x_ = 1.676 Mg m^−^^3^
Monoclinic, *P*2_1_/*c*	Mo *K*α radiation, λ = 0.71073 Å
Hall symbol: -P 2ybc	Cell parameters from 4608 reflections
*a* = 9.7863 (7) Å	θ = 2.2–28.3°
*b* = 11.3715 (8) Å	µ = 1.64 mm^−^^1^
*c* = 7.3462 (5) Å	*T* = 100 K
β = 109.1495 (8)°	Block, colorless
*V* = 772.28 (9) Å^3^	0.25 × 0.20 × 0.15 mm
*Z* = 2	

### Data collection

**Table:**

Bruker SMART APEX diffractometer	1773 independent reflections
Radiation source: fine-focus sealed tube	1634 reflections with *I* > 2σ(*I*)
graphite	*R*_int_ = 0.026
ω scans	θ_max_ = 27.5°, θ_min_ = 2.2°
Absorption correction: multi-scan (*SADABS*; Sheldrick, 1996)	*h* = −12→12
*T*_min_ = 0.685, *T*_max_ = 0.792	*k* = −14→14
7174 measured reflections	*l* = −9→9

### Refinement

**Table:**

Refinement on *F*^2^	Primary atom site location: structure-invariant direct methods
Least-squares matrix: full	Secondary atom site location: difference Fourier map
*R*[*F*^2^ > 2σ(*F*^2^)] = 0.021	Hydrogen site location: inferred from neighbouring sites
*wR*(*F*^2^) = 0.062	H atoms treated by a mixture of independent and constrained refinement
*S* = 1.12	*w* = 1/[σ^2^(*F*_o_^2^) + (0.0283*P*)^2^ + 0.4612*P*] where *P* = (*F*_o_^2^ + 2*F*_c_^2^)/3
1773 reflections	(Δ/σ)_max_ = 0.001
114 parameters	Δρ_max_ = 0.43 e Å^−^^3^
2 restraints	Δρ_min_ = −0.35 e Å^−^^3^

### Fractional atomic coordinates and isotropic or equivalent isotropic displacement parameters (Å^2^)

**Table:**

	*x*	*y*	*z*	*U*_iso_*/*U*_eq_	
Zn1	0.5000	0.5000	0.5000	0.00889 (9)	
O1	0.45689 (11)	0.33271 (9)	0.56937 (15)	0.0132 (2)	
O2	0.28013 (11)	0.21247 (9)	0.57458 (17)	0.0167 (2)	
O3	0.51490 (11)	0.55292 (9)	0.78390 (15)	0.0129 (2)	
H3	0.564 (2)	0.6126 (14)	0.831 (3)	0.030 (6)*	
O4	0.00548 (11)	0.66106 (11)	0.01696 (15)	0.0160 (2)	
H4	−0.0808 (12)	0.678 (2)	−0.009 (3)	0.040 (7)*	
N1	0.27253 (13)	0.52389 (11)	0.44766 (18)	0.0095 (2)	
C1	0.32624 (16)	0.31061 (13)	0.5430 (2)	0.0116 (3)	
C2	0.21180 (15)	0.40649 (12)	0.4649 (2)	0.0114 (3)	
H2A	0.1466	0.3821	0.3363	0.014*	
H2B	0.1528	0.4130	0.5511	0.014*	
C3	0.26823 (16)	0.60594 (13)	0.6035 (2)	0.0128 (3)	
H3A	0.1690	0.6082	0.6106	0.015*	
H3B	0.2935	0.6862	0.5732	0.015*	
C4	0.37309 (16)	0.56760 (14)	0.7966 (2)	0.0141 (3)	
H4A	0.3757	0.6275	0.8955	0.017*	
H4B	0.3400	0.4925	0.8360	0.017*	
C5	0.20539 (15)	0.57679 (13)	0.2541 (2)	0.0114 (3)	
H5A	0.2129	0.5197	0.1561	0.014*	
H5B	0.2620	0.6472	0.2442	0.014*	
C6	0.04786 (16)	0.61242 (13)	0.2055 (2)	0.0131 (3)	
H6A	−0.0125	0.5431	0.2091	0.016*	
H6B	0.0370	0.6713	0.2990	0.016*	

### Atomic displacement parameters (Å^2^)

**Table:**

	*U*^11^	*U*^22^	*U*^33^	*U*^12^	*U*^13^	*U*^23^
Zn1	0.00708 (13)	0.00885 (13)	0.01018 (13)	−0.00067 (8)	0.00204 (9)	0.00051 (8)
O1	0.0092 (5)	0.0119 (5)	0.0173 (5)	−0.0003 (4)	0.0026 (4)	0.0023 (4)
O2	0.0106 (5)	0.0123 (5)	0.0236 (6)	−0.0021 (4)	0.0008 (4)	0.0057 (4)
O3	0.0102 (5)	0.0136 (5)	0.0133 (5)	−0.0013 (4)	0.0019 (4)	−0.0020 (4)
O4	0.0111 (5)	0.0223 (6)	0.0127 (5)	0.0045 (4)	0.0014 (4)	0.0056 (4)
N1	0.0094 (6)	0.0089 (5)	0.0098 (6)	−0.0002 (4)	0.0026 (5)	0.0004 (4)
C1	0.0120 (7)	0.0118 (6)	0.0100 (6)	0.0003 (5)	0.0021 (5)	0.0005 (5)
C2	0.0091 (7)	0.0114 (6)	0.0131 (7)	−0.0011 (5)	0.0029 (5)	0.0013 (5)
C3	0.0125 (7)	0.0127 (6)	0.0129 (7)	0.0015 (5)	0.0037 (6)	−0.0013 (5)
C4	0.0124 (7)	0.0184 (7)	0.0117 (7)	0.0001 (5)	0.0042 (6)	−0.0013 (6)
C5	0.0092 (7)	0.0133 (7)	0.0109 (7)	0.0007 (5)	0.0021 (5)	0.0016 (5)
C6	0.0106 (7)	0.0151 (7)	0.0118 (7)	0.0022 (5)	0.0012 (5)	0.0016 (5)

### Geometric parameters (Å, °)

**Table:**

Zn1—O1	2.0488 (10)	N1—C3	1.4882 (18)
Zn1—O1^i^	2.0488 (10)	C1—C2	1.533 (2)
Zn1—O3^i^	2.1292 (11)	C2—H2A	0.9900
Zn1—O3	2.1292 (11)	C2—H2B	0.9900
Zn1—N1^i^	2.1484 (13)	C3—C4	1.516 (2)
Zn1—N1	2.1484 (13)	C3—H3A	0.9900
O1—C1	1.2542 (18)	C3—H3B	0.9900
O2—C1	1.2537 (18)	C4—H4A	0.9900
O3—C4	1.4308 (18)	C4—H4B	0.9900
O3—H3	0.84 (1)	C5—C6	1.519 (2)
O4—C6	1.4212 (17)	C5—H5A	0.9900
O4—H4	0.83 (1)	C5—H5B	0.9900
N1—C2	1.4836 (18)	C6—H6A	0.9900
N1—C5	1.4839 (18)	C6—H6B	0.9900
			
O1—Zn1—O1^i^	180.0	N1—C2—H2A	108.7
O1—Zn1—O3^i^	91.59 (4)	C1—C2—H2A	108.7
O1^i^—Zn1—O3^i^	88.41 (4)	N1—C2—H2B	108.7
O1—Zn1—O3	88.41 (4)	C1—C2—H2B	108.7
O1^i^—Zn1—O3	91.59 (4)	H2A—C2—H2B	107.6
O3^i^—Zn1—O3	180.0	N1—C3—C4	110.98 (12)
O1—Zn1—N1^i^	97.12 (4)	N1—C3—H3A	109.4
O1^i^—Zn1—N1^i^	82.88 (4)	C4—C3—H3A	109.4
O3^i^—Zn1—N1^i^	82.74 (4)	N1—C3—H3B	109.4
O3—Zn1—N1^i^	97.26 (4)	C4—C3—H3B	109.4
O1—Zn1—N1	82.88 (4)	H3A—C3—H3B	108.0
O1^i^—Zn1—N1	97.12 (4)	O3—C4—C3	110.24 (12)
O3^i^—Zn1—N1	97.26 (4)	O3—C4—H4A	109.6
O3—Zn1—N1	82.74 (4)	C3—C4—H4A	109.6
N1^i^—Zn1—N1	180.0	O3—C4—H4B	109.6
C1—O1—Zn1	115.48 (9)	C3—C4—H4B	109.6
C4—O3—Zn1	109.91 (8)	H4A—C4—H4B	108.1
C4—O3—H3	108.3 (16)	N1—C5—C6	115.44 (12)
Zn1—O3—H3	118.6 (16)	N1—C5—H5A	108.4
C6—O4—H4	105.4 (17)	C6—C5—H5A	108.4
C2—N1—C5	112.49 (11)	N1—C5—H5B	108.4
C2—N1—C3	112.67 (11)	C6—C5—H5B	108.4
C5—N1—C3	111.57 (11)	H5A—C5—H5B	107.5
C2—N1—Zn1	106.95 (8)	O4—C6—C5	106.39 (12)
C5—N1—Zn1	109.26 (9)	O4—C6—H6A	110.5
C3—N1—Zn1	103.34 (9)	C5—C6—H6A	110.5
O2—C1—O1	124.08 (13)	O4—C6—H6B	110.5
O2—C1—C2	116.06 (13)	C5—C6—H6B	110.5
O1—C1—C2	119.86 (13)	H6A—C6—H6B	108.6
N1—C2—C1	114.08 (12)		
			
O3^i^—Zn1—O1—C1	−92.62 (10)	O3^i^—Zn1—N1—C3	−157.45 (8)
O3—Zn1—O1—C1	87.38 (10)	O3—Zn1—N1—C3	22.55 (8)
N1^i^—Zn1—O1—C1	−175.50 (10)	Zn1—O1—C1—O2	178.78 (12)
N1—Zn1—O1—C1	4.50 (10)	Zn1—O1—C1—C2	−0.37 (17)
O1—Zn1—O3—C4	−78.86 (9)	C5—N1—C2—C1	128.95 (13)
O1^i^—Zn1—O3—C4	101.14 (9)	C3—N1—C2—C1	−103.88 (14)
N1^i^—Zn1—O3—C4	−175.83 (9)	Zn1—N1—C2—C1	8.99 (14)
N1—Zn1—O3—C4	4.17 (9)	O2—C1—C2—N1	174.38 (12)
O1—Zn1—N1—C2	−7.26 (9)	O1—C1—C2—N1	−6.4 (2)
O1^i^—Zn1—N1—C2	172.74 (9)	C2—N1—C3—C4	68.96 (15)
O3^i^—Zn1—N1—C2	83.44 (9)	C5—N1—C3—C4	−163.38 (12)
O3—Zn1—N1—C2	−96.56 (9)	Zn1—N1—C3—C4	−46.10 (13)
O1—Zn1—N1—C5	−129.27 (9)	Zn1—O3—C4—C3	−30.45 (14)
O1^i^—Zn1—N1—C5	50.73 (9)	N1—C3—C4—O3	53.60 (16)
O3^i^—Zn1—N1—C5	−38.57 (9)	C2—N1—C5—C6	68.31 (16)
O3—Zn1—N1—C5	141.43 (9)	C3—N1—C5—C6	−59.44 (16)
O1—Zn1—N1—C3	111.84 (9)	Zn1—N1—C5—C6	−173.08 (10)
O1^i^—Zn1—N1—C3	−68.16 (9)	N1—C5—C6—O4	179.67 (11)

Symmetry codes: (i) −*x*+1, −*y*+1, −*z*+1.

### Hydrogen-bond geometry (Å, °)

**Table:**

*D*—H···*A*	*D*—H	H···*A*	*D*···*A*	*D*—H···*A*
O3—H3···O2^ii^	0.84 (1)	1.85 (1)	2.651 (2)	161 (2)
O4—H4···O2^iii^	0.83 (1)	1.89 (1)	2.715 (2)	178 (3)

Symmetry codes: (ii) −*x*+1, *y*+1/2, −*z*+3/2; (iii) −*x*, *y*+1/2, −*z*+1/2.
